# Dispensers under the Insurance Act

**Published:** 1912-06-22

**Authors:** Geo. W. Udale

**Affiliations:** Pharmaceutical Chemist. "Ellastone," Harlington, Middlesex


*To the Editor of* THE HOSPITAL.

### "Dispensers under the Insurance Act."

*To the Editor of* THE HOSPITAL.

SIR,—It was not my intention to enter into a polemic or forensic correspondence in respect of the above; I was merely desirous of pointing out that the position of the dispenser *quâ* dispenser is not, nor ever has been, one on which there is, or was, any misunderstanding. The position has always been thoroughly understood by all whose association work has brought them in direct contact with dispensers throughout the country. Perhaps I may be "beating the air" (not for the first time), but I adhere to that which I have written, together with its inferences, and in the absence of the necessary authoritative regulations my intelligent anticipation was equally in harmony with your contributor's forecast. Unconsciously, perhaps, your editorial footnote supports my original contention, and as I am more or less disinterested in the final judgment, I can afford, as you suggest, "to wait and see." With compliments.—Yours faithfully,

GEO. W. UDALE, M.P.S.,
Pharmaceutical Chemist.

"Ellastone," Harlington, Middlesex, June 15.

